# Editorial: Recent trends and advancements in multispectral and hyperspectral imaging for cancer detection

**DOI:** 10.3389/fimmu.2025.1752197

**Published:** 2025-12-08

**Authors:** I-Chen Wu, Hsiang-Chen Wang, Arvind Mukundan

**Affiliations:** 1 Division of Gastroenterology, Kaohsiung Medical University Hospital, Kaohsiung Medical University, Kaohsiung, Taiwan; 2 Biomedical Artificial Intelligence Academy, Kaohsiung Medical University, Kaohsiung, Taiwan; 3 Department of Mechanical Engineering, National Chung Cheng University, Chia Yi, Taiwan; 4 School of Engineering and Technology, Sanjivani University, Sanjivani Factory, Kopargaon, Maharashtra, India

**Keywords:** hyperspectral imaging, multispectral imaging, deep learning, cancer detection, radiomics, oncology

Hyperspectral imaging (HSI) acquire images throughout numerous spectral bands, encompassing visible wavelengths (380 nm–700 nm), near-infrared (800 nm–2500 nm), and mid-infrared (2500 nm–15000 nm) (1, 2). Each pixel contains several spectral bands to obtain specific information regarding certain pollutants (3). HSI acquires a data cube that encompasses 1D spectral and 2D spatial information (4, 5). A one-dimensional spectrum illustrates the absorption of light by tissues at a pixel across various wavelengths, while two-dimensional vectors denote each pixel’s position within the spectral spatial array (6). HSI has been employed in numerous biomedical applications to assess the physical properties of intricate surfaces and identify cancer cells through precise spectral signatures (7). The conventional RGB technique employs solely three primary colors by amalgamating varying light intensity (8). Simultaneously, multispectral imaging (MSI) is based on RGB and captures 3 to 10 non-contiguous spectral bands within the electromagnetic spectrum (9). HSI surpasses all other broadband images by offering more comprehensive information. Recent improvements in multispectral and hyperspectral imaging provide a solution by delivering high-resolution spectral data that can reveal biochemical and morphological alterations suggestive of early-stage cancer. This Research Topic, “Recent Trends and Advancements in Multispectral and Hyperspectral Imaging for Cancer Detection,” was created to aggregate pioneering research and comprehensive reviews that seek to tackle the significant challenge of prompt and precise cancer detection, vital for enhancing patient outcomes.

Li et al. conducted a comparative evaluation of 95 BI-RADS 4 breast lesions utilizing conventional ultrasonography, contrast-enhanced ultrasound (CEUS), and shear wave elastography (SWE). Both CEUS and SWE exhibited significant diagnostic sensitivity, specificity, and accuracy when compared to US alone. The diagnostic performance enhanced further when modalities were combined, with the US + CEUS + SWE achieving the greatest AUC (0.946) and total accuracy (94.7%). The triple-modality approach enhances differentiation between benign and malignant tumors and reduces unnecessary biopsies.

The research by Guo et al. presents an uncommon case of primary adrenal melanoma in a 37-year-old man who presented with back pain and rapidly developed widespread metastases. A CT scan identified a heterogeneous tumor in the adrenal glands, and a sample confirmed the presence of melanoma, exhibiting positive staining for Melan-A, HMB-45, and SOX-10. No primary lesion was found outside the adrenal glands. The paper analyzes diagnostic characteristics, differentiates between PAM and pigmented pheochromocytoma, and discusses treatment, emphasizing the importance of meticulous evaluation and pathological diagnosis for accuracy.

Hao et al. propose a deep learning-based system for medical image analysis applicable to multispectral and hyperspectral images, aimed at improving cancer detection. The approach tackles challenges associated with high-dimensional data and limited annotations using multi-scale feature extraction, attention processes, domain adaptation, and self-supervised learning. Predictions are enhanced via a knowledge-guided regularization module. Experimental evidence demonstrates that the framework exhibits superior accuracy, robustness, and interpretability compared to existing models, hence indicating its potential to significantly improve AI-based diagnostics with the extensive data offered by spectral imaging.

Tang et al. assessed the capability of CT radiomics, in conjunction with clinical characteristics, to distinguish between focal organizing pneumonia (FOP) and peripheral lung cancer (PLC). Sixty FOP and sixty PLC patients were examined. Logistic regression identified pleural adhesion, outer lung zone involvement, liquefactive necrosis/cavity formation, and extensive spiculation as independent risk factors for FOP. CT radiomics (AUC = 0.859) and clinical characteristics (AUC = 0.895) demonstrated substantial diagnostic efficacy, whereas their combined model exhibited superior performance (AUC = 0.955), greatly surpassing the results of each individual test.

Geng et al. evaluated a multi-task deep learning network (MTDL) utilizing abdominal CT to identify early clinically relevant prostate cancer. A 3D U-Net model was built and evaluated utilizing data from 539 patients and was compared with radiomics and single-task deep learning models. The MTDL nomogram, integrating CT predictions with age and PSAD, had a high AUC across training, test, and validation cohorts, outperforming other models. Results indicate that multi-task deep learning utilizing abdominal CT possesses significant potential for the early detection of prostate cancer.

Wang et al. compared an AI system integrating YOLO and HSI to improve the first detection of superficial esophageal neoplasms. The model utilized 1,836 endoscopic pictures for training and over 500 endoscopic images for validation to categorize normal tissue, dysplasia, and squamous cell carcinoma. The accuracy of RGB-based WLI and NBI was 0.83 and 0.82, respectively, while HSI-based models achieved 0.90 and 0.89. Overall, HSI enhanced diagnostic accuracy by 8% and significantly improved the diagnosis of esophageal cancer by the utilization of AI.

Liang et al. developed an MRI-based nomogram integrating clinical data, handmade radiomics, and 2.5D deep learning radiomics (DLR) features to distinguish between benign and malignant spinal compression fractures. Conventional radiomics and deep learning models shown similarity among 234 patients; however, the integrated DLR models, especially the ExtraTrees-based DLR, demonstrated superior diagnosis accuracy. The integration of clinical and MRI characteristics significantly improved performance, with the DLR nomogram achieving the highest AUC. The proposed multimodal approach serves as a significant instrument for improving VCF distinction in clinical practice.
