# Correction: Editorial: Recent trends and advancements in multispectral and hyperspectral imaging for cancer detection

**DOI:** 10.3389/fimmu.2026.1798272

**Published:** 2026-02-05

**Authors:** I-Chen Wu, Hsiang-Chen Wang, Arvind Mukundan

**Affiliations:** 1 Division of Gastroenterology, Kaohsiung Medical University Hospital, Kaohsiung Medical University, Kaohsiung, Taiwan; 2 Biomedical Artificial Intelligence Academy, Kaohsiung Medical University, Kaohsiung, Taiwan; 3 Department of Mechanical Engineering, National Chung Cheng University, Chia Yi, Taiwan; 4 School of Engineering and Technology, Sanjivani University, Sanjivani Factory, Kopargaon, Maharashtra, India

**Keywords:** hyperspectral imaging, multispectral imaging, deep learning, cancer detection, radiomics, oncology

Affiliation “Biomedical Artificial Intelligence Academy, Kaohsiung Medical University, Kaohsiung, Taiwan” was omitted for author I-Chen Wu. This affiliation has now been added for author I-Chen Wu.

As a result, the affiliation numbering and author affiliation assignments have been updated accordingly (I-Chen Wu: 1,2; Hsiang-Chen Wang: 3; Arvind Mukundan: 3,4*).

The funders National Science and Technology Council, the Republic of China (NSTC 113-2221-E-194-011-MY3) and Kaohsiung Medical University (KMU-TC114A07-1) were erroneously omitted.

The original version of this article has been updated.

